# A toolkit for new facioscapulohumeral muscular dystrophy trial sites

**DOI:** 10.1177/22143602251399244

**Published:** 2025-12-26

**Authors:** Joost Kools, Lawrence Korngut, Janet Petrillo Ballantyne, Irene Roozen, Ria de Haas, Amanda Hill, Teresinha Evangelista, Valeria A Sansone, Richard Roxburgh, Hanns Lochmuller, Jeff Statland, Nicholas E Johnson, Nicol Voermans

**Affiliations:** 1 Department of Neurology, Donders Institute for Brain, Cognition and Behaviour, 6034 Radboud University Medical Center, Nijmegen, the Netherlands; 2 Department of Clinical Neurosciences, 70401 Hotchkiss Brain Institute, University of Calgary, Calgary, Canada; 3 Department of Rehabilitation, Donders Institute for Brain, Cognition and Behaviour, Amalia Children's Hospital, 6034 Radboud University Medical Center, Nijmegen, the Netherlands; 4 FSHD Europe - The European Voice for People with FSHD, the Netherlands; 5 FSHD Society - Empowering FSHD Patients, Accelerating FSHD Progress, USA; 6 Institute of Myology, Nord/Est/Ile-de-France Neuromuscular Reference Center, Pitié-Salpêtrière Hospital, APHP, 72755 Sorbonne University, Paris, France; 7 The NEMO Clinical Center, Neurorehabilitation Unit, 9338 University of Milan, Milan, Italy; 8 Neurology Department, 62710 Auckland City Hospital, Te Toka Tumai, Te Whatu Ora Health New Zealand, Auckland, New Zealand; 9 Children's Hospital of Eastern Ontario Research Institute, Division of Neurology, Department of Medicine, The Ottawa Hospital, Brain and Mind Research Institute, University of Ottawa, Ottawa; 10 Department of Neurology, 21638 University of Kansas Medical Center, Kansas City, Kansas, USA; 11 Department of Neurology, Virginia Commonwealth University, Richmond, VA, USA

**Keywords:** FSHD, facioscapulohumeral muscular dystrophy, trial, trial readiness, trial unit, management

## Abstract

Numerous potential treatments are being developed for facioscapulohumeral muscular dystrophy (FSHD). Project Mercury was initiated to overcome challenges that could slow or prevent effective therapies from widespread availability to patients. It is important that upcoming trials include trial sites from different countries. We share our lessons learnt in clinical trials to assist inexperienced sites to become eligible for upcoming clinical trials. To become an eligible site, several key elements need to be in place such as personnel, facilities, and accessible patient populations. Clinical trial networks, patient advocacy groups and patient registries can support new sites in establishing these elements. As the preparation, execution and close-out of clinical trials generally involve the same steps every time, it is recommended to create and follow a trial roadmap. Most clinical trials are sponsor-initiated and involve working closely with the sponsor and vendors. It is therefore important to understand each other perspectives and goals for each trial. Once a drug receives regulatory approval and becomes available for market use new challenges arise such as patient reimbursement and phase 4 surveillance of the patients. In summary, we are at a pivotal time for FSHD and other rare neuromuscular disorders with the development of new disease modifying therapies. It is vital that as many sites as possible can participate in upcoming trials.

## Introduction

**Figure 1.**
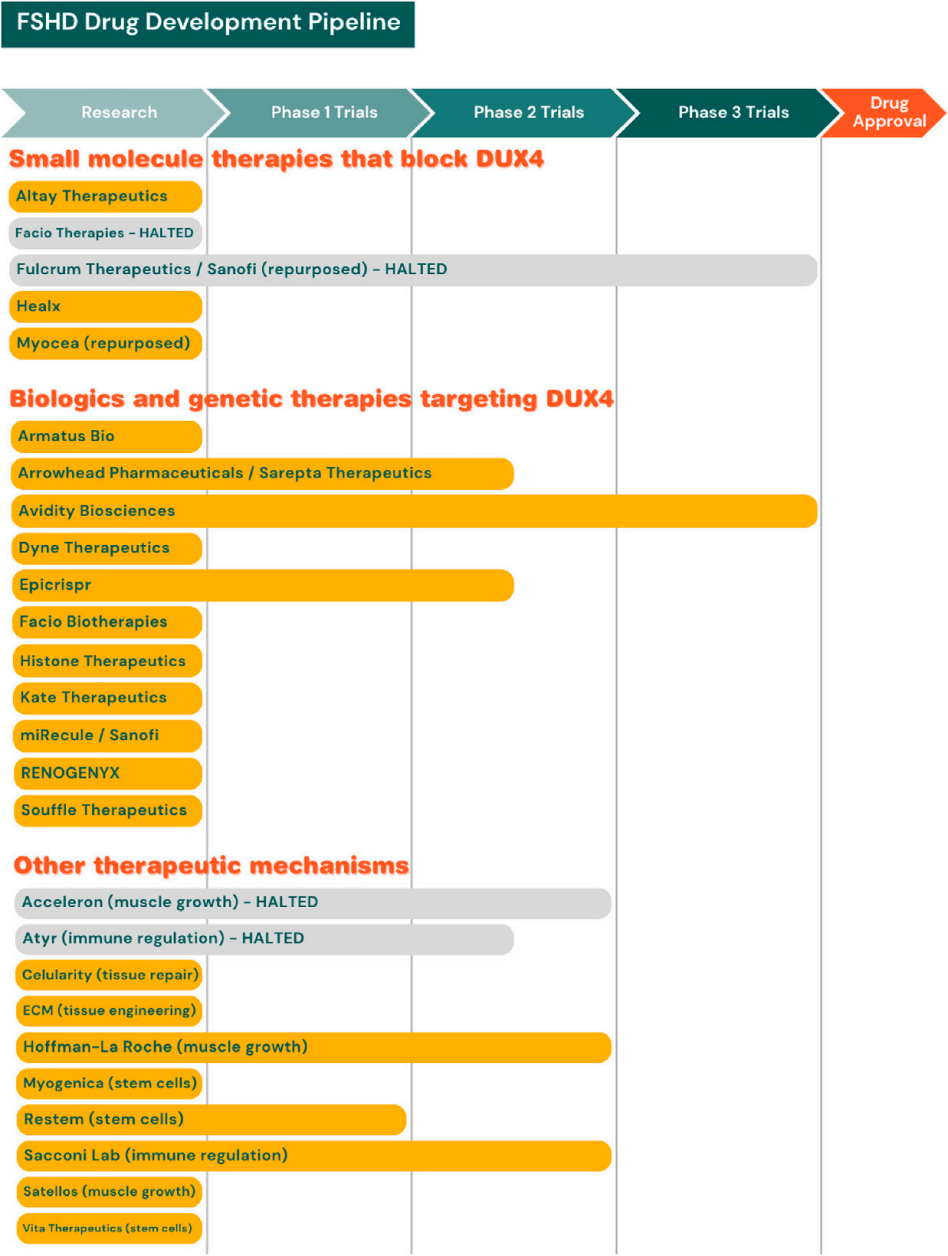
FSHD drug development pipeline.^2^

In recent years, the number of clinical trials for facioscapulohumeral muscular dystrophy (FSHD) has increased, with numerous potential treatments currently under development (Figure 1).^1^ Trials in rare diseases like FSHD have many challenges, among which is the need to efficiently initiate multiple sites in different countries to be able to achieve the recruitment targets and consequently be able to address the country specific clinical trial regulations. To optimize the feasibility of upcoming trials, clinical researchers have organized themselves in international networks (e.g., FSHD Clinical Trial and Research Network (CTRN) in the USA and FSHD European Trial Network (ETN)). However, to ensure that upcoming trials and treatments are accessible to FSHD patients worldwide, it is important for less experienced sites in other regions to start participating in FSHD trials.

The FSHD Society, the world's largest research-focused patient advocacy organization for FSHD, has launched an initiative called Project Mercury to facilitate the growth of expertise in less experienced centers and encourage their participation in FSHD trials.^3^ Project Mercury is an open collaboration among stakeholders worldwide; aiming to overcome the challenges that could slow or prevent effective therapies from becoming widely available to patients. This collaboration takes place at the global level through a Global Task Force and at the local level, through Country Working Groups. The Global Task Force and the Country Working Groups are all led by patient advocacy organizations of the World FSHD Alliance. Project Mercury aims to build a global cohort of 10,000 clinical-trial-ready, well-characterized patients, expand and optimize the world-wide clinical trial infrastructure; and prepare to overcome the barriers that delay patient access to therapies once approved.

To optimize world-wide the clinical trial infrastructure, we will share the lessons learnt from previous FSHD clinical trials. This will help less experienced trial sites in engaging with the FSHD research networks, develop clinical trial infrastructure, and conduct trials effectively. We divided the lessons learnt in four larger sections: Preparation, Trial Roadmap, Sponsor Perspective and Regulatory approval and patient access. While this paper was primarily focused on FSHD clinical trials, many of the lessons learned are applicable to other rare (neuromuscular) diseases.

## Approach

The concept of creating this trial hub roadmap design evolved during a meeting of Project Mercury in Banff, Canada, in November 2023. One of the authors (LK) presented his experience of the neuromuscular trial site in Calgary, Canada, including current site best practices (from the perspective of investigators), current site challenges (from the perspectives of investigators, industry, and patients), components of a FSHD Hub Toolkit and existing resources available. An FSHD clinical trial group consisting of clinical researchers of trial sites involved in the Mercury project (USA, Canada, the Netherlands) was installed. Three online meetings took place to discuss the topic list, which led to the initial draft of this manuscript. Next, the manuscript was reviewed and rewritten by many experts in the field until consensus was reached.

Although the manuscript was written with a sponsor-initiated clinical trial in mind, most of the lessons learnt are applicable to investigator-initiated trials or natural history studies with some adjustments. Topics will include the necessary trial site infrastructure, lessons learned from conducting trials, clinical trials from the sponsor's perspective, and drug accessibility following regulatory approval.

## Preparation

Sites should have several key elements in place (e.g., personnel, facilities, and accessible patient populations) before they can be deemed eligible to participate in clinical trials. The existing clinical trial networks (Table 1), patient advocacy groups and registries can support new sites in establishing these elements.

**Table 1.** International networks of expertise for neuromuscular diseases (NMDs) in general, and for FSHD in particular. This non-exhaustive list highlights key networks currently active in the neuromuscular field, especially in FSHD. It includes both networks of clinicians and patient organizations, which play a crucial role in the execution of ongoing trials and the establishment of new trial sites.

Network	Region	Description
*International networks of expertise for NMDs in general*		
NMD4C 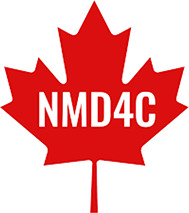	Canada	The Neuromuscular Disease Network for Canada (NMD4C) is the pan-Canadian network that brings together the country's leading clinical, scientific, technical, and patient expertise to improve care, research, and collaboration in neuromuscular disease.
MDA Care Center Network 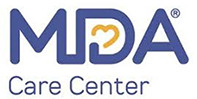	USA	Located at more than 150 of the top health care institutions across the United States, MDA Care Centers and Affiliates serve as the nexus for multidisciplinary neuromuscular care and medical research.
EURO-NMD 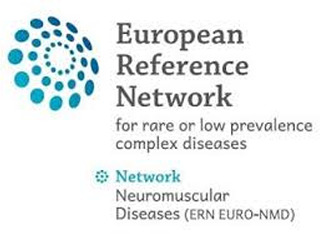	Europe	EURO-NMD is one of the 24 European Reference Networks (ERNs). It aims to harmonize and implement standards for clinical and diagnostic best practice, improving equity of care provision, decreasing time to diagnosis, increasing cost efficiency, access to specialist training and education, application of eHealth services, development and application of care guidelines, facilitating translational and clinical research, harmonizing data and samples for research reuse, and sharing of high-quality data.
ANN 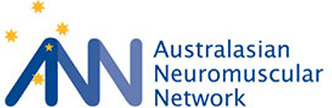	Australia	The Australian neuromuscular network aims to be a coordinated and collaborative voice at a national level to advocate for improved funding for diagnostic services, registers and clinical trials infrastructure. We can achieve our vision by establishing a cohesive, integrated neuromuscular network which enables people to work together across Australia and New Zealand, for the well-being of patients.
NMS ANZ 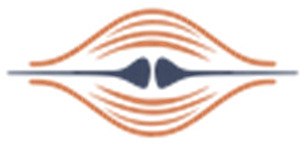	New-Zealand - Australia	Neuromuscular Society of Australia and New Zealand (NMSANZ) is dedicated to promoting research, education, and advocacy for those affected by neuromuscular diseases
Japan Muscular Dystrophy Association 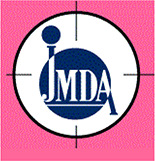	Japan	The Japan Muscular Dystrophy Association aims to promote research to establish the pathogenesis of and find effective treatment for muscular dystrophy, and to provide medical care to the patients and their families so that they can maintain a good quality of life.
NMTI-SA 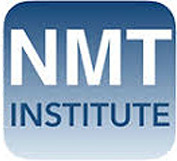	South-America	NeuroMuscular Taping South America Institutehas been created to reach out to other countries in the South and Central American region. The NMTI-SA Institute will offer training, educational programs, research development, volunteer projects based on the NMT Therapeutic Concept. Trainings are available to a multidisciplinary medical professional group.
MDFSA 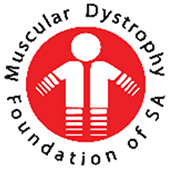	South Africa	The MDFSA mission is to support people affected by muscular dystrophy and neuromuscular disorders and endeavour to improve the quality of life of the patients in South-Africa.
TREAT-NMD 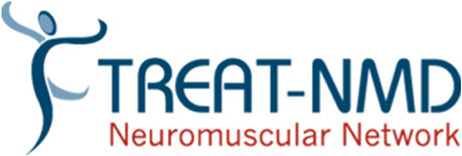	Global	TREAT-NMD is a global network of experts in the neuromuscular field. It aims to operate a collaborative, inclusive global network and organisational infrastructure that will overcome fragmentation, providing support services, information and data to advance treatment, diagnosis and care for neuromuscular patients globally.
*International networks of expertise for FSHD specific*		
FSHD Society 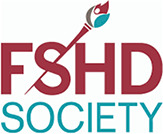	North-America and global	The FSHD Society's goal for all impacted by FSHD is two-fold: 1) Speed the delivery of effective treatments and a cure and; 2) Ensure those impacted have what they need to live their best life. The FSHD Society has played a key role in the advancement of FSHD therapies and provides support to our community worldwide.
FSHD Europe 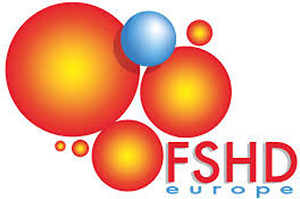	Europe	The aim of FSHD Europe is to act as a catalyst, working with all stakeholders to promote joint work around FSHD that will enable the faster development of treatments, and make them accessible to people with FSHD.
FSHD Global 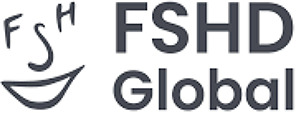	Australia	The FSHD Global Research Foundation focuses on finding treatments and a cure for FSHD. In doing so, we fund world class medical research, awareness and education.

### Personnel

Since most of the upcoming trials are likely to follow a randomized, double-blind, placebo-controlled design, the required personnel at the site can be divided in three groups: the clinical team, the pharmacy team, and the biotech (or research) team. At minimum the clinical team consists of three roles: a principal investigator (PI), a trial coordinator and a research nurse. Sub-investigators and physiotherapists are often required, either due to protocol mandates or to ensure the smooth execution of the trial. The pharmacy team will require both blinded and unblinded personnel. The biotech team differs per protocol and may require personnel to process blood samples (some sites allow research nurses to process sampling), perform MRIs or process muscle biopsy samples.

The PI is the main point of contact regarding medical issues, performs physician-related tasks during the trial (e.g., evaluate adverse events, perform physical examination) and is ultimately responsible for the quality of the study (Box 1 provides tips for new clinical investigators). The trial coordinator negotiates and manages the budget, oversees the logistical preparations of the trial and supports the submission of regulatory documents. The research nurse performs nurse-related tasks during the trial (e.g., draw blood samples, make EKGs and measure vital parameters), coordinates the scheduling with the participant and is in charge of data management. In addition, some sites allow research nurses and study coordinators to process sampling.

Box 1: Tips for new clinical investigators.

- Become familiar with Principal Investigator responsibilities reviewing ICH GCP requiring protection of the rights, safety and welfare of study participants and ensure appropriate regulations are followed while conducting the trial.
- Budget: thoroughly review and understand the protocol and study manuals to ensure that the study budget is calculated appropriately. Recommendations include costing both per subject and individual invoiceable fees, rejecting withhold if requested, and to request advance payment for at least 1 or more completed participant up front along with start-up fees. The start-up fees will be non-refundable (try to negotiate a higher start-up fee especially), but some or all of one participant to full completion could be refunded if the visits don’t occur.
- Carefully review payment terms confirming reasonable timeframes.
- Initially hire a part time research coordinator and increase time to full-time as funds permit. Typically, institutions will require salary for length of the contract, but there may be opportunities for hourly/casual staff initially.
- Have your research coordinator connect with professional societies so they are up to date with regulatory requirements and have opportunities for additional support.
- Schedule introductory meetings with legal, ethics, finance, pharmacy, human resources and lab departments and begin building relationships at institution.
- Ensure you have site Standard Operating Procedures in alignment with ICH GCP and appropriate regulations.
- Network with patient groups, colleagues involved in clinical trials, other trial sites.
- Ensure you check in on the financial health of your clinical trial several times a year. Check that invoices have been submitted to sponsor for payment, that payments are received, and that expenses are appropriate (staff salary, lab, pharmacy, materials & supplies).
- Ensure team members and referring physicians are aware you are recruiting for your trial. In-services provides an opportunity to discuss the study and patient population considered for the trial.
- Look into the possibility of department or institution support along with external grants. See if your organization can share a research coordinator with your trial.
- Be mindful that Sponsors and Clinical Research Organizations (CROs) expect that the Principal Investigator understands their responsibilities, including source, research charts/EMR, and investigator site files. Although occasionally a Clinical Research Associate (CRA or monitor) will be quite helpful to a site/PI, the CRO is delegated to certain sponsor responsibilities, not site support.
- Know that there will undoubtedly be challenges during the lifetime of a clinical trial and that this is expected even with experienced sites. With the support of your research staff, departments and institution along with communicating to the sponsor and CRO, the experience can be very meaningful and have great impact to study participants regardless of study outcome.

In our experience, every role within a trial team is crucial, and developing and maintaining expertise in these roles will benefit the sites. Several factors promote an effective working relationship within the team, including clear communication on setting of priorities, availability of investigators to the team for urgent issues, signatures, and study visits, as well as mentorship and support for career development. It is crucial for investigators to foster a positive work environment by handling their staff with respect and consistently acknowledging their contributions. Furthermore, PIs should be aware and react accordingly to the (mental) health status of their team members as burnout symptoms are common, which reduces team effectiveness and increases risk of sudden resignations resulting in higher turnover.

Finally, the principal investigator's availability can be the rate limiting step for enrollment in a clinical trial, especially if they are the only physician on the study team. Hence, the PI, as other team members, must ensure that they have sufficient protected time and scheduling flexibility to accommodate the study visit schedule with infrequent visits that often need to occur within narrow time windows.

### Facilities

An appropriate facility is essential for smooth execution of trial visits, maintain a tight time schedule and adhere to Good Clinical Practice (GCP) guidelines.^4^ Ideally, the facilities are strategically located in close proximity to one another in order to minimize travel time for the participant. This is crucial to reduce fatigue in less mobile patients and optimize the reliability of any physical measurements. Physical exam rooms need to be spacious enough to contain an exam table (preferably with the quantitative muscle assessment equipment built around it as this is often used in neuromuscular trials), the Reachable Workspace (RWS) set-up (including the 2-meter space between the RWS equipment and the chair) and a closet to store smaller equipment like a handheld dynamometer. Furthermore, a lockable archive to store trial master files and any paper-based source material is required. Drug trials require that any documentation is archived for at least 25 years, we advise to keep the documents archived locally for the first year in case of any audits or inspections and use an external facility for long-term archiving afterwards to minimize the necessary storage facility. We strongly recommend using electronic data management instead of paper-based sources, so long as the electronic system adheres to the regulations. In general, most electronic systems in hospitals contain an audit trail to track any changes and adhere to the privacy laws. If there are any doubts, we advise discussing this with the Contract Research Organization (CRO) involved, the sponsor, or national regulatory boards.

### Recruitment and communication plan

Quick recruitment of study participants lowers the overall cost of studies and helps sponsors meet their strict timelines, which is crucial in their competitive industry. Therefore, sites that can support this process are more appealing to sponsors. Several measures can be implemented to accelerate enrollment at a site, such as establishing a patient registry, engaging an active patient advocacy group, and creating a clear communication plan. It has become more common to recruit patients for FSHD trials by inviting potential eligible patients via national patient registries.^5–7^ The Updated TREAT-NMD core dataset for FSHD enables the selection of patients based on genotype and phenotype.^8^ Generally, registry-based randomized controlled trials offer several advantages over conventional randomized trials, including lower costs, enhanced generalizability of findings, faster consecutive enrollment, and the potential for complete follow-up of the reference population.^9^ Well-organized registries can also be used to conduct natural history studies to validate outcome measures, particularly patient-reported outcome measures.^7,10,11^

If utilizing an FSHD-specific registry is not feasible due to financial or personnel restraints, European centers can make use of the European EURO-NMD registry platform.^12^ It can facilitate efficient patient recruitment for clinical trials by providing access to a large, diverse cohort of individuals with FSHD. Furthermore, patient advocacy groups (or a small number of patient representatives) can contribute to the dissemination of information about upcoming trials and informed consents. Examples of possible communication pathways are webinars, patient conferences, (secured) social media groups and newsletters. Regardless of the communication form, it is important to have a clear communication plan in place. Patients appreciate knowing when and where to find information on clinical trials. Sites will benefit because an established plan reduces workload and increases the chance of being eligible for trials from the Sponsor's perspective.

### Patient education and preparation

Participating in a clinical trial is different from a clinical visit to a neuromuscular center. Patients must receive comprehensive education on both the benefits and challenges of participating in the trial. Education can be provided by the neuromuscular networks, trial sites and patient organizations. It should be readily available, easily accessible and available in different formats (e.g., print, video, web).

Regardless of the format, we suggest to provide training on the following topics: the general pathway and timelines of an investigational product (IP) from preclinical to marketing phase; the rationale behind a randomized, double-blind, placebo-controlled trial and the likely possibility for joining an open-label extension phase; the commonly used outcome measures (e.g., MRI, RWS, strength measurements and muscle biopsies); the most frequently applied eligibility criteria (e.g., age, no comorbidities, moderately affected patients and genetic confirmation) to prevent disappointment for (pre)screen failures;, and lastly the burdens accompanying trial participation (e.g., physical burden due to physical testing or psychological burdens) need to be discussed, so patients can make a well-considered decision.^13,14^

Besides education, sites can take several preparatory steps for patients who aim to participate in a clinical trial. Most of the clinical trials will require a relatively recent genetic confirmation from an accredited lab using standardized methods.^15,16^ If these tests are not available at a national level, international networks can provide the solution. The recent best practice guidelines provide a list of accredited labs for FSHD diagnostic testing.^16^ General health is also a key factor in determining eligibility. Sites should support patients in maintaining a healthy weight, conduct annual tests to monitor heart, liver, and kidney function, and ensure effective management of any comorbidities with stable medication dosages. Additionally, pharmaceutical treatments tend to be more effective in patients who receive optimal symptomatic care for their FSHD symptoms. Therefore, we recommend that all sites follow the most recent evidence-based guidelines when treating FSHD patients.^17,18^

Lastly, it might be beneficial for the participant and researcher to discuss in advance what they are hoping to get out of the trial. Where possible the participant should provide an objective metric to indicate the success of the therapeutic. There are multiple scales that may be used, but the most widely disseminated is the goal attainment scale.^19^

## Trial roadmap

**Figure 2.**
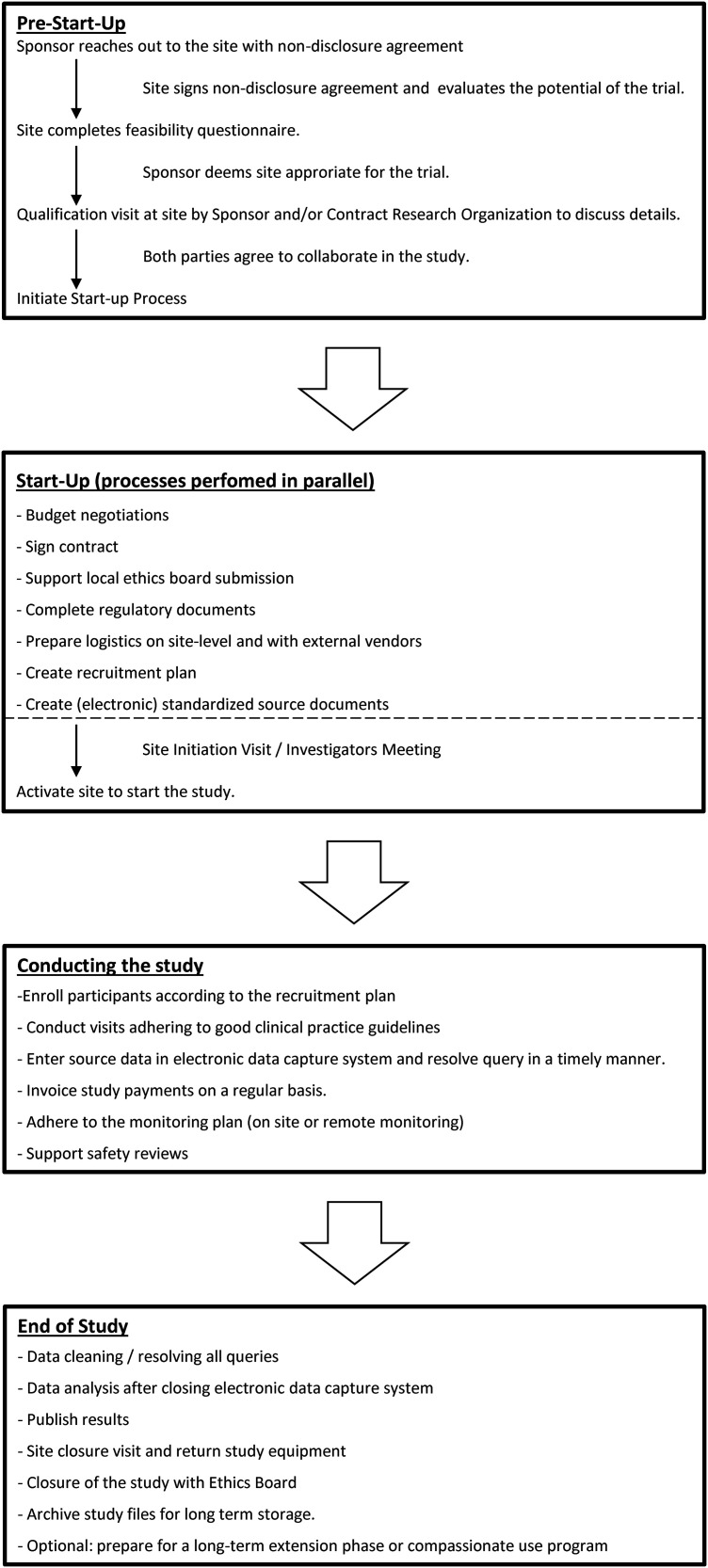
Preparation: the trial path. The preparation, execution and close-out of a trial require the same steps every time.

The preparation, execution and close-out of a trial generally involve the same key steps each time (Figure 2). It is time efficient, reduces error and improves the quality if the team adheres to the same roadmap when a new trial is introduced. This trial path can be modeled similarly to care pathways for rare diseases.

### Initiation

Drawing from our experience, we have a few additional recommendations. When completing a feasibility questionnaire for sponsors, provide data-driven responses from your clinic database regarding overall patient availability, if accessible. Ensure accuracy in reporting patient numbers and potential recruitment rates and avoid making unrealistic commitments. FSHD Europe is collaborating with the Care and Trial Site Registry (CTSR: https://ctsr.uniklinik-freiburg.de/ctsr/), an online database that tracks site-level information relevant to clinical studies.^20^ Sponsors can use this database to quickly identify promising trial sites and their facilities. Furthermore, consider the PI's time, staffing resources, and do not take on too many trials at once. It is better to execute one trial exceptionally well, with strong recruitment and site metrics, than to underperform across multiple protocols. Keeping the startup process efficient and smooth builds a good relationship with the sponsor from the start. Careful estimates of timelines will help the sponsor and CRO in ensuring timely site initiation. Appropriate budget assessments are key. If the site is operating at a loss or just breaking even, growth becomes impossible. Staff may need to be released after a study concludes, which can disrupt continuity for future trials or limit opportunities for program expansion. We recommend creating a study budgeting template or using a commercially available tool. When multiple sites are involved in a single country, it may be beneficial to collaborate during negotiations, although costs can vary between centers. If desired, a lead site can share the (electronic) source documents created for the clinical trial.

Currently, feasibility assessments are typically conducted solely by sponsors, requiring trial sites to complete a questionnaire and undergo a qualification visit to outline, among other factors, their facilities, patient population, and recruitment methods. Given the increasing number of trial options in FSHD, it may be valuable for sites or research networks to formally assess the feasibility of the sponsor. A feasibility assessment could include clear statements on data transparency and ownership, an agreed-upon publication plan regardless of the results, a commitment to making placebo data available for future studies, and the availability of open-label extensions programs. For example, the losmapimod program was discontinued after the phase 3 results showed no additional benefit of losmapimod compared to placebo. Fulcrum Therapeutics is currently transferring the data to the FSHD Society to ensure the data will remain available to advance our knowledge of FSHD and clinical trials.^21^ We commend Fulcrum Therapeutics on this course of action and highly encourage all pharmaceutical companies to follow this example.

The ethical approval process varies between and even within countries. Sponsors appreciate the individual sites knowledge around their timelines and process. In the United States, many sites can use a commercial or other central Institutional Review Board (IRB). While there is wide variation in the time it takes to cede the initial review to the central IRB, this process significantly reduces subsequent amendments to the ethical approval documents. Recently, a centralized process was introduced in Europe to enhance the efficiency of obtaining ethical approval across EU countries. As of January 31 2023, the Clinical Trials Information System (CTIS) is the single-entry point for submission of data and information relating to clinical trials.^22^ This marks a significant shift from the previous situation, where authorization had to be requested separately in each European Member States. Furthermore, within each Member State, a submission needs to be made to a National Competent Authority and one or multiple ethics committees. The processes for these submissions differ from one Member State to the other.

### Execution

During trial execution it is important to adhere to all GCP regulations of and other ethics requirements of national regulatory bodies. PIs should provide support and ensure that new staff members are trained correctly. University medical centers generally provide GCP training and certification at local or national level. Furthermore, the TREAT-NMD Global Network has been successfully delivering educational masterclass programs about neuromuscular disorders since 2015.^23^ EURO-NMD offers a wide range of webinars on various neuromuscular topics. Furthermore, The FSHD Society is developing continuing medical education programs based on the new care guidelines, as part of Project Mercury. Collectively, these efforts are expected to expand knowledge of FSHD and its symptomatic management among a broad group of clinicians.

The required trainings will differ per clinical trial and vendors used, but the following procedures often require qualifications in FSHD trials: muscle biopsies, performing and processing whole body MRIs and several clinical outcome assessments (RWS, Motor Function Measure, Handheld Dynamometry and Qualitative Muscle Assessments). We recommend carefully keeping track of these qualifications to prevent unnecessary repetition of training procedures.

Furthermore, the PI should be aware of the responsibilities, experience, training, and qualifications of each site staff member to whom tasks are delegated. It's crucial that every staff member understands their responsibilities. To build trust through effective task delegation, it's essential to ensure proper supervision and the PI's availability.

Especially for inexperienced sites, we recommend fully enrolling the first participant and reviewing the process before enrolling additional patients, as some procedures may not yet be fully established. This small time-out before scheduling new screening visits, allows a site to finetune these processes which will prevent unnecessary challenges. Having two staff members in the lead for one trial will minimize the number of careless protocol deviations and ensures continuity. Trials usually involve a lot of training on several systems, equipment, and outcomes measures. Keeping track of these training sessions in Investigator Site Files is essential and may prove beneficial for future trials as this might give exemption for future training. It is also helpful to keep track of platforms, logins, and passwords for each trial to ensure easy access to the required platforms and prevent disruptions in access. Box 2 provides additional tips for trial execution. Box 3 provides recommendations for future trials.

Box 2: Ten tips for FSHD trial execution.

- Involve patient representatives in the team that plans the trial. Develop a communication plan for the whole process
- Organize a webinar or on-site meeting at the launch of the trial to inform all possible trial participants.
- Inform the general practitioner and other health care provides by a letter to be distributed by the patient.
- Know your patients and make sure they know your team: Have the patients registered at your site as clinical patient and provide an emergency card.
- Make the facility ‘FSHD-accessible’: elevators, chairs with armrests, non-slippery floor, examination table with adjustable height.
- Patients have expressed what they think is feasible for MRI Scans: 30 min, then have a break to be able to move and get out of scanner. 2^nd^ session is possible.^24^
- Renal function should be assessed with cystatin-C instead of creatininein case of muscle wasting.
- Incomplete right bundle branch block is known to occur in FSHD patients, but is usually benign, asymptomatic and does not require follow-up.
- Assess the experiences of participants systematically and prospectively.
- Ensure data and privacy protection towards the sponsor (e.g., the sponsor mustn’t be given identifiable data of the participants) and explain to participants how their social media activities can adversely affect the trial and ask that they not post details.

Box 3: Recommendations for future FSHD trials.

- Enable routes for participation for both drug- and trial naïve patients and patients that have been exposed to study drugs and/or patients that are using one or more newly registered drugs.
- Widen the inclusion criteria: also include pre-symptomatic patients, wheelchair-dependent patients, patients with D4Z4 repeat size 10.
- Include Goal Attainment Scaling (GAS) as a personalized patient-reported outcome measure.^48^
- Assess the experiences of the trial participants systematically and prospectively as a quality check.^14^
- Increase patient involvement in trial design and execution.
- Include home monitoring of motor function as part of decentralized trials.^49^
- Ensure optimal symptomatic treatment including physical training before recruitment in a trial.
- Combine different therapeutic approaches (in complex trial designs). Box 3 provides recommendations for future trials.

Given certain eligibility criteria, it is crucial that patients are pre-screened to avoid unnecessary screen failures. It is also important to inform non-selected patients about the trial's overall design and eligibility criteria. Many registry participants may learn about the trial through patient organizations or social media. Providing timely and clear information about why a patient was not selected can help prevent confusion and frustration. Not receiving an invitation or experiencing a pre-screen failure can be disappointing and have a significant emotional impact on patients, especially considering that, at present, there is no disease-modifying therapy for FSHD, and trial participation is often perceived as hope for a potential treatment. Nevertheless, it is essential for patients to remain hopeful,engaged and well-informed for future trials. The message should be that participation in a clinical trial does not guarantee therapeutic benefit. Randomization to placebo, to an ineffective investigational drug, or to a compound with unforeseen adverse effects is at least as likely as allocation to an effective treatment. Clear communication, support, and alternative options are vital for these patients, which can be offered by site personnel or patient advocacy groups.

### Diversity and inclusivity

A huge challenge for clinical trials in neuromuscular disorders is sampling bias. Sampling bias limits the generalizability of findings because it is a threat to external validity, specifically population validity. Racial and ethnic minorities continue to be underrepresented in neuromuscular and other clinical trials. Although barriers to diversity in trials are well recognized, sustainable solutions for overcoming them have proved elusive.^25^ Ramdharry presented a key note lecture on the importance of engaging patients from diverse backgrounds in neuromuscular research at the WMS in 2023.^26^ Including patients from different ethnic groups requires a broader scope at inclusion. Diverse recruitment approaches are required since patient registries are also likely to be biased. The patient advocacy groups are likely to be able to play an important role in this. Furthermore, the National Institute of Health (NIH) has a dedicated institute that aims to increase the inclusivity in clinical trial research within the National Institute on Minority Health and Health Disparities (NIMHD). It is essential to have a wide range of people from different communities participate in clinical trials to reduce biases, promote social justice and health equity, and produce more innovative science. The NIMHD's mission is therefore to lead scientific research to improve minority health and reduce health disparities.^27^ One factor expected to increase the diversity of trial participants is remote trial design, which will reduce the barriers posed by in-person visits for less mobile patients.^28^

### Trial close-out

After a trial concludes, it is crucial to share the knowledge gained (regardless of the outcome) with the academic community and patient groups. This should be done orally (presentations, webinars) and in text (articles, websites of patient organizations, newsletters, and peer-reviewed publication in a scientific journal). Patient advocacy groups play a key role in translating academic language into terms that are accessible to the public. Trial participants might require care after the trial for ongoing adverse events, psychological support, or physical therapy. Ensure that the participants are followed-up either within your clinic or their regular specialist. Inform the general practitioner and other health care providers about end of study.

### Compassionate use programs

Compassionate use programs enable access to experimental therapies for patients with advanced or rapidly progressive disease who are ineligible for clinical trials. These programs offer critical real-world insights into treatment safety, tolerability, and functional outcomes, thereby complementing formal studies and accelerating the clinical development of potential therapies for rare diseases. No major sponsors of FSHD therapeutics currently operate an established compassionate-use or expanded-access program, but several maintain general expanded-access policies and will evaluate requests case-by-case.

## Sponsor perspective

Most clinical trials would not have been possible without the investments of the pharmaceutical companies. It is important to consider the sponsor as a valuable partner. It is crucial to understand each other's goals and decisions during the partnership. Therefore, we aimed to elucidate clinical trials from the sponsor's perspective, which is not always agreeable with the clinician's perspective, and give recommendations to Sponsors to improve the partnership with trials sites.

### Definition of successful trial

Sites may define a successful trial based on a high retention rate and the number of participants who can remain in the open-label extension phase after the trial concludes. From the sponsor's perspective, trial success is defined as a positive trial outcome (e.g., meeting efficacy endpoints) in the planned time. Furthermore, each sponsor has a vested financial interest in the successful conduct of a clinical trial. Most companies have limited resources to to navigate their drug through the commercialization process. They will likely prioritize fast enrollment and efficient trial designs. In addition to these financial considerations, they have to adhere to the regulatory responsibilities regarding the safe conduct of the study and ensuring high quality data. In this context, sponsors in fields like FSHD can benefit from well-established academic research networks that have already engaged patients, developed clinical endpoints, and identified high-performing sites.

### Trial design

**Figure 3.**
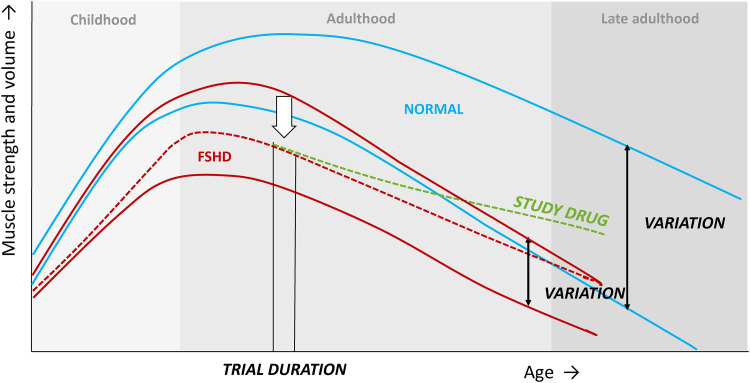
A schematic overview to show one of the challenges when designing an FSHD clinical trial.

Compared to other neuromuscular disorders, FSHD generally has a prolonged progression over the lifetime. It can takes years for significant changes to occur in patients using current measurement tools.^7,29^ We currently do not expect new therapies to drastically improve the disease state of patients, but rather that they will slow the disease progression (Figure 3). This combination of prolonged disease progression and minor improvements by new interventions makes it challenging to find statistically significant changes in a clinical trial with usually a duration of 1–2 years. One way to overcome this challenge is by using surrogate biomarker which are used as substitutions for a direct measure of how a patient feels, functions or survives.^30^ Surrogate biomarkers should be sensitive to change and correlate well with clinical outcome assessments or patient reported outcome measures. In the case of FSHD, fat fraction and lean muscle volume on whole body MRI are often used as surrogate biomarkers.^31^ Additionally, DUX4 concentration in muscle tissue can be used as this is the main molecular driver of the disease, but DUX4 is difficult to detect reliably and requires an invasive muscle biopsy procedure.^32^ Recently, Avidity Biosciences presented that they may have found a reliable molecular blood biomarker, but details on this biomarker are not yet shared due to the pending patent.^33^ Using surrogate biomarkers enables faster development of new therapies, but also come with a caveat. Phase 4 surveillance studies are most likely mandatory after market approval to study the long-term effect of the drug on actual clinical outcomes in the patients.

The blue lines show the progression of muscle strength and its variation in the healthy population. The red lines show the progression of muscle strength and its variation in the FSHD population: there is overlap with the graph of the population without FSHD. The red dotted line is that of an imaginary patient, participation in a trial. The green dotted line shows that we currently expect new therapies to slow disease progression, not immediately improve the disease state. The two black lines show the relatively short trial duration. The green and red line show minimal differences during the trial, which visualizes the difficulty of showing a statistically significant difference in an FSHD trial.

Another challenge sponsors face in rare conditions like FSHD is that many aspects of the genetics, clinical endpoints, and participant selection are investigated contemporaneously. This places a heavy reliance on key opinion leaders to provide accurate information about trial design.

The first consideration in trial design relates to mitigating the safety risks inherent to therapeutic trials. This often means that the eligibility criteria will focus on identifying individuals without other serious medical conditions, especially those affecting the heart, liver, and kidneys depending on the individual therapy. Along with this, many regulatory agencies require sponsors to start development in adults who have the capacity to understand these risks. Other considerations around inclusion criteria in FSHD include which modes of genetic testing, severity of D4Z4 repeat array contraction and if FSHD type 2 are accepted, as these factors may affect the participant's disease severity and course.^16^

One of the most promising developments in recent years is the ability to detect disease activity in specific muscles by whole body MRI. Along with criteria around functional status (e.g., ambulatory) these efforts have allowed sponsors to identify a more homogenous population for clinical trials. Since minimizing sample size is often a consideration in early phase dose escalation studies, it is important to limit the variation between participants in an inherently variable condition. One challenge that remains is the ability to detect functional changes in a highly heterogenous condition during the short duration of a trial. While there continues to be refinement in outcome measures to allow for earlier detection of efficacy (e.g., the RWS is recently developed and was used as the primary outcome measure in the losmapimod phase 3 trial^34^), this remains a challenge throughout all trial phases in FSHD.

While Sponsors should consider a homogenous adult population in phase 1 and 2 clinical trials, different considerations exist for phase 3 and 4 studies. It is crucial that the therapy is studied in as diverse a population as possible to ensure broad accessibility to all patients in the event of market approval. This includes the ability to understand the safety and efficacy in pediatric studies. Beyond including children, sponsors should also consider the inclusion of underrepresented minority populations and non-ambulatory individuals. In the draft FDA guidance, several strategies may be deployed.^35^ First, sponsors can pre-specify their efficacy population, which may be a subset of all of those included in the clinical trial. This allows for the inclusion of a wider range of severity. Second, sponsors should consider selecting study sites with higher populations of underrepresented minorities. Third, sponsors should consider minimizing unnecessary endpoints or visits to reduce the overall burden on participants, which will make the trial more accessible for all patients. Finally, sponsors should actively consider their participant reimbursement process to minimize the financial barrier to participate in a trial.

**Figure 4.**
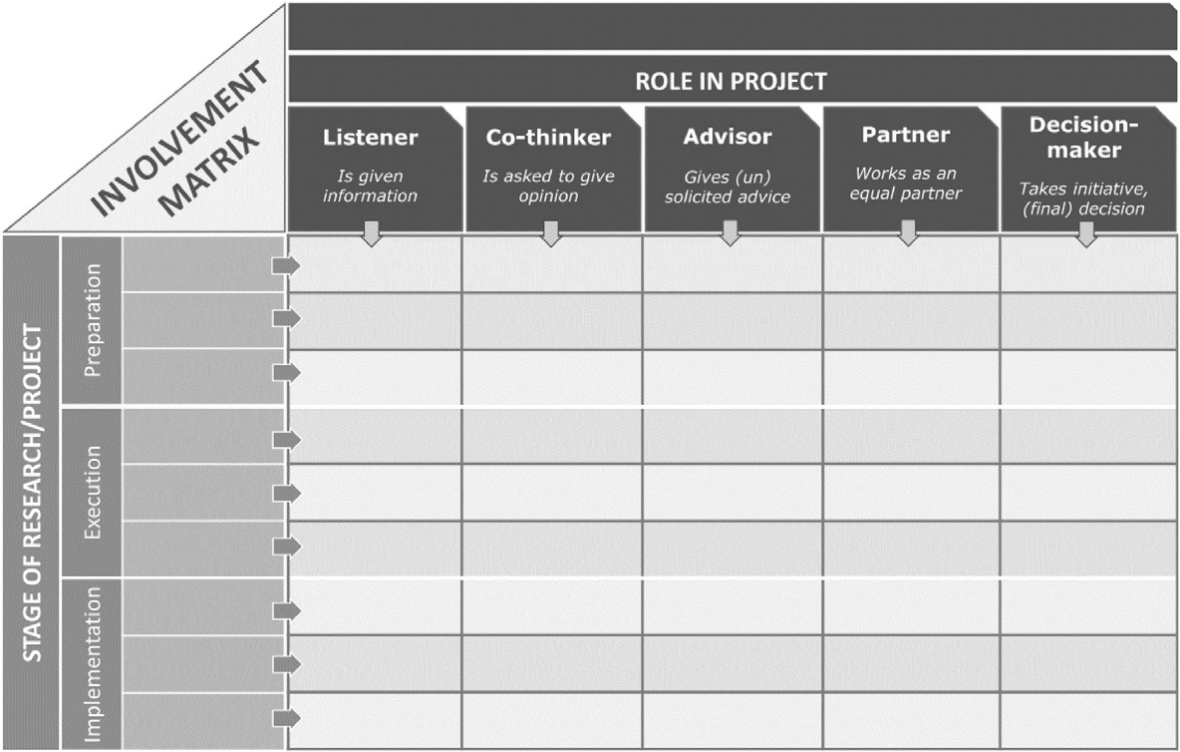
The involvement matrix: this tool has been developed to promote collaboration with patients (from the age of 12) in projects and research. It is a tool for project leaders and clinical researchers. It aims to dialogue with the patient about the role the patient wishes to play in a project. The various roles of involvement are shown horizontally. The phases of a project are shown vertically. The proposed main phases are ‘preparation’, ‘execution’ and ‘implementation’, but these can be further specified by the user. Combining the roles and phases results in a matrix containing cells. Involvement Matrix; www.kcrutrecht.nl/involvement-matrix. ^©^ Center of Excellence for Rehabilitation Medicine Utrecht, used with permission.^39^

We recommend Sponsors involve patient representatives in the conceptualization process of trials, which is becoming more common.^36^ Umbrella organizations, such as FSHD Europe, often provide a community advisory board to advise and consult with sponsors. Involving patient representatives early in trial process will be helpful in determining the feasibility and burden of the study protocol, choosing appropriate outcome assessments, and ensuring the patient information is understandable for laymen. These different ways in which patients can contribute to clinical research are summarized in the Involvement matrix (Figure 4). Two European organizations for rare diseases, Eurordis (Eurordis Open Academy) and Eupati (EUPATI open classroom) provide free in-depth courses for patients to get involved in clinical research as a patient representative and to take a leading role in engaging with pharmaceutical companies and regulatory agencies.^37,38^ These courses provide the knowledge to actively engage in study preparations, design, performance, analysis, and dissemination of the results. Sites and patient advocacy groups can encourage patients to follow these courses and become an active and well-informed patient representative.

### Communication

Sponsors have a privileged position in the drug development process. For many individuals living with FSHD, the condition is relentlessly progressive in themselves and their family members. Sponsors should therefore consider early and frequent communications with the patient community to set realistic expectations. Sponsors can work with both the sites and patient advocacy groups to ensure patients are aware of the time required to move through clinical development.^36^

Establishing regular communication or touchpoints with site investigators can help reduce the volume of email exchanges. These meetings are important to develop and maintain the relationship with the site investigators. In many instances, site investigators are trying to balance multiple research, clinical and educational demands. Efficient communication will be rewarded and may foster enrollment in the study. Site investigators and their study coordinators will prioritize work with a sponsor they have developed a good relationship with, improving both recruitment and retention. Besides sponsor communication, streamlining the communications with CROs is also of vital importance. In general, almost every person on a delegation receives emails from the CRO, which makes it difficult to determine which emails are important and which can be discarded. A form of ‘hourglass’ communication would be ideal, where all communications from the CRO are collected by one person in the organization, send to one person at the site, who will then redistribute the information to the necessary site personnel.

Turnover both at the Sponsor and the CRO often impairs these communication channels and relationships. One strategy to reduce this risk is to ensure back-ups for the individuals involved in face-to-face communications. If one of the individuals leaves the company, the second individual can serve as a warm transition to the individual new to the role. One of the frustrations investigators often voice, particularly with CROs, is the repeated education of the new staff on the disease. Staff should consider reviewing some basic information about the condition prior to engaging with sites to make these interactions as productive as possible.

Sponsors and their CROs should have a plan around the development of Suspected Unexpected Serious Adverse Reaction (SUSARs) and consider practicing their roles should this develop in the clinical trial. The plan should be shared with investigators so that all can operate efficiently and without error in the event this occurs. Sponsors should proactively discuss the need to obtain specimens in the event of a SUSAR so that appropriate determinations around relatedness can be obtained. Additionally, a communication plan regarding an (unexpected) halt of trial should be in place. Most of the pharmaceutical companies are public companies which prohibits them from updating sites upfront about results and possible halts. It is therefore crucial that communication in such events is clear and swift, ensuring all participants are updated as quickly as possible.

## Regulatory approval and patient access

### Regulatory approval (marketing authorization)

The centralized procedure is the primary pathway for obtaining marketing authorization for medicines that are intended to be marketed in all EU Member States, as well as Iceland, Norway, and Liechtenstein. Under this procedure, the European Medicines Agency (EMA) evaluates the safety, efficacy, and quality of the medicinal product. This procedure is mandatory for certain types of medicines, including orphan drugs. If the product meets the necessary standards, EMA grants a single market authorization, which is valid across all these countries. This central approval ensures that a product can be marketed and sold throughout the EU without needing separate approvals from each individual member state

### Pricing and reimbursement approval

After market approval, it can take a significant amount of time before the treatment becomes available to patients in all countries. Each EU country uses its own Health Technology Assessment (HTA) agency to determine if the drug is cost-effective, its price and reimbursement conditions. Because FSHD is a rare neuromuscular disease, regulatory authorities and HTA bodies may not be automatically familiar with the disease. Continental and national patient organizations, together with the healthcare professionals, play an important role in explaining and educating all stakeholders about the FSHD disease landscape, including disease symptoms, patient populations, progression, standard of care, impact on quality of life and the ability to work or attend school (impact on society).^40^ Even when reimbursement is approved, it may not be available for the complete patient population due to evidence-based coverage decisions. Reimbursement restrictions (e.g., age, subtype of the disease, disease progression) vary between and even within countries leading to situations that are hard to explain to patients and can be very difficult to accept, especially when the drug might be available just across the border a couple of miles away.

The OdySMA initiative of SMA Europe provides an excellent example of improving access: it maps, visualizes and centralizes knowledge and data around access to treatments for spinal muscular atrophy (SMA) across Europe.^41^ It shows that 18 countries fail to provide available life-changing medicine without restrictions. It is important to realize that trial design (e.g., eligibility criteria) can greatly impact treatment accessibility in different countries. Therefore, involving the patient community, HTA bodies and other access stakeholders in the early phase of drug development and trial design can be very helpful to improve accessibility post-marketing. Importantly, it is essential to collaborate with national patient organizations in communicating with the patient community to manage expectations, not only for patients themselves, but also for their families and friends.

After market approval, once the therapy is available and reimbursed, it is often necessary to monitor its safety and efficacy in real-world use through post-marketing surveillance, due to uncertainties about clinical evidence and budget impact. For this purpose, well-structured patient registries and clinically relevant outcome measures (for adults and children) are of great importance. There are methodological challenges to developing and using real-world evidence for HTA decisions, specifically for rare diseases, such as differences in data quality, outcome measures, use of historical controls and small numbers of patients.^42^ Well-structured registries are therefore essential not only at the start of drug development but remain crucial beyond market access to collect long-term post marketing data.^43,44^ Additionally, sites may require a change in their (neuromuscular) clinical program and/or require more personnel to be able to dose and monitor all the patients.

## Future perspectives

The number of trials in FSHD is increasing. At the 2024 FSHD Society's Connect Conference, eight pharmaceutical companies participated in a panel discussion on ongoing or future trials. The number of FSHD trial participants is estimated at a few thousand in the next five years. Project Mercury aims to improve the availability of well-characterized and diverse patients in both disease state and geographically.^45^ It is becoming more common to involve patient representatives in the early stages of trial design, to ensure the voice of the patients is accounted for.^46^ Finally, we expect that use of digital outcome measures for motor function outside a clinical setting is feasible and will be employed in a broad range of rare neuromuscular diseases. Future research should focus on validation of devices, variables, and algorithms to allow for regulatory qualification and widespread adoption.^47^

## Conclusion

In summary, FSHD, and other rare genetic disorders, are at a pivotal time whereby molecularly targeted therapies have the potential to modify the course of an otherwise relentlessly progressive disease. It is incumbent on the ecosystem of academic sites, supporting organizations, and sponsors to devise efficient methods to conduct these interventional trials on behalf of the participants in them. This roadmap may act as a guide, for those involved, to consider when designing and implementing clinical trials.
